# Exciton Binding Energy of Monolayer WS_2_

**DOI:** 10.1038/srep09218

**Published:** 2015-03-18

**Authors:** Bairen Zhu, Xi Chen, Xiaodong Cui

**Affiliations:** 1 Physics Department, University of Hong Kong, Hong Kong, China

## Abstract

The optical properties of monolayer transition metal dichalcogenides (TMDC) feature prominent excitonic natures. Here we report an experimental approach to measuring the exciton binding energy of monolayer WS_2_ with linear differential transmission spectroscopy and two-photon photoluminescence excitation spectroscopy (TP-PLE). TP-PLE measurements show the exciton binding energy of 0.71 ± 0.01 eV around K valley in the Brillouin zone.

Coulomb interactions are significantly enhanced in low dimensional systems as a result of spatial confinement and reduced Coulomb screening. Consequently, excitons, quasiparticles of electron-hole pairs bounded by Coulomb force, play a pronounced role in their optical aspects. A few paradigms of the pronounced excitonic effects have been demonstrated in quantum dots and carbon nanotubes, where the exciton binding energies are found to be a fraction of their band gaps in these quasi-zero dimensional (0D) and one dimensional (1D) systems^1^,^2^,^3^. Prominent exciton effects are also widely expected in intrinsic 2D systems, for instance, monolayer crystals of transition metal dichalcogenides (TMDCs) owing to the reduced dielectric screening and spatial confinements^4^,^5^,^6^,^7^,^8^. Monolayer TMDC is an intrinsic 2D crystal consisting of two hexagonal planes of chalcogen atoms and an intermediate hexagonal plane of metal atoms in a prismatic unit cell. Particularly MX_2_ (MoS_2_, MoSe_2_, WS_2_ and WSe_2_) experiences a transition from indirect gap in bulk form to direct gap of visible frequency in monolayer, where the band gap is located at K (K′) valley of the Brillouin zone^9^,^10^,^11^,^12^. *Ab initio* calculations show the direct-gap exciton binding energy in the range of 0.5–1 eV, which is around 1/3–1/2 of the corresponding optical direct gap^4^,^5^,^6^,^7^,^8^. The modulated absorption/reflection spectroscopy shows the binding energy of direct gap exciton is around 55 meV in bulk crystals^13^. Such a big exciton binding energy in bulk form guarantees the robust excitonic nature of optical properties in ultrathin counterparts. Furthermore, photoluminescence (PL) experiments identify electron (hole)-bounded excitons, so called trions, with a charging energy *E_bX−_* of 18 meV, 30 meV and 20–40 meV in monolayer MoS_2_, MoSe_2_ and WS_2_, respectively^14^,^15^,^16^. With a simple 2D exciton model, one could estimate the exciton binding energy around 10 times that of the trion, if equal effective electron's and hole's mass are assumed^17^. However, the direct measurement of the exciton binding energy in monolayer TMDC is lacking (After posting of this work, the experimental studies^18^,^19^,^20^,^21^,^22^,^23^,^24^ on exciton binding energy and exciton excited states in monolayer TMDCs have been reported).

Here we report experimental approaches to measuring the exciton binding energy of monolayer WS_2_ with linear differential transmission spectroscopy and two-photon photoluminescence excitation spectroscopy (TP-PLE). The TP-PLE resolves the excited states of excitons and the interband transition continuum. The exciton binding energy of 0.71 ± 0.01 eV of the band-edge excitons around K valley in the Brillouin zone is extracted by the energy difference between the ground state exciton and the onset of the interband continuum.

Monolayer WS_2_ was mechanically exfoliated from single crystal WS_2_ and identified with optical microscopes and photoluminescence spectroscopy (supplementary information). The samples in differential transmission measurements were made by transferring from silicon substrates to freshly cleaved mica substrates as described in Ref. 25. All other measurements were performed on WS_2_ monolayers on silicon substrates with a 300 nm oxide cap layer. The electric-gate dependent PL measurements were conducted with a back-gated field effect transistor structure with Au electrodes. The TP-PLE spectroscopy was undertaken at ambient conditions by deploying a confocal setup with a 20× achromatic objective and a Ti:sapphire oscillator (80 MHz, 100 fs).

Figure 1(a) summarizes linear optical measurements of monolayer and multilayer WS_2_. There are distinct peaks in the differential transmission spectra, labeled as “A”, “B” and “C”, respectively^12^,^13^. Peaks “A” around 2.02 eV and “B” around 2.4 eV at room temperature present the excitonic absorptions of the direct gap located at K valley of the Brillouin zone. The separation between “A” and “B” of 0.38 eV rising from the splitting of the valence band minimum (VBM) due to the spin-orbit coupling (SOC) at K (K′) valley is almost constant in all the layers with various thickness, which is consistent with the PL spectra^11^,^12^. It is the direct result of the suppression of interlayer coupling at K (K′) valley owing to the giant SOC and spin-valley coupling in tungsten TMDC with 2*H* stacking order, in which each unit layer is a π rotation of its adjacent layers^11^. The peak “C” around 2.8 eV was recognized as the excitonic transitions from multiple points near Γ point of the Brillouin zone^5^,^12^. Unlike in many semiconductors, the linear absorption spectra of WS_2_ display no gap between distinct excitons and the continuum of the interband transitions. In addition to the overlap with the higher-energy states (exciton C), the continuous absorption may also originate from the strong electron (hole)-phonon coupling and the superposition of high-energy excited states of excitons (exciton A and exciton B) that fill the gap between the ground state excitons and the interband continuum in the linear absorption spectra^5^,^19^. As the temperature drops to 10 K, peak “A” and “B” are both blue-shifted by around 0.1 eV and peak “C” is shifted by 0.06 eV as shown in Figure 1(c). The difference of the blue-shift is the direct consequence of the diverse locations of the excitons in the Brillouin zone: exciton “A” and “B” are formed at K valley while “C” is around Γ point. Nevertheless, the continuous absorption still survives and no distinct single-particle band edge emerges at cryogenic temperature (10 K). It is difficult to resolve the exciton binding energy by the linear absorption spectra owing to the overlap of high-energy excitons (exciton C), the strong electron-phonon coupling and the transfer of the oscillator strength from interband continuum to exciton states. The strong electron-phonon coupling and the transfer of the oscillator strength are the direct consequences of enhanced coulomb interactions in low dimensional materials as reflected in carbon nanotubes^2^,^26^.

Figure 1(b) shows the absorbance of atomically thin WS_2_ films as a function of the thickness above their ground exciton energy, which is approximated with the differential transmission. The absorbance of monolayers and multilayers is linearly proportional to their thickness, each layer absorbing around 2.0% and 3.4% at excitations of 2.088 eV and 2.331 eV, respectively. The linear layer dependence of the absorption gives an experimental evidence of the suppression of interlayer hopping in 2*H* stacked WS_2_ as a result of spin-valley coupling^11^,^27^. The thickness dependence could also be used as a thickness monitor for multilayer characterization. There is a side band at the red side of exciton A, which modifies the lineshape away from the symmetric Lorentzian or Gaussian shape. We tentatively attribute the side band to the contribution of electron/hole-bound excitons or trions^14^,^15^,^16^. Although the monolayer WS_2_ is not intentionally doped, the structural defects and substrate effects such as charge transfer and defects modulate the carrier density away from its insulating state. To confirm the origin of the side band around exciton A, we record the PL spectra of monolayer WS_2_ at various electric gating (from 70 V to −70 V) at room temperature in vacuum, which continuously tunes the Fermi level of monolayer WS_2_ as illustrated in Figure 2(a).

There is a prominent peak X^−^ at the red side of the free exciton X^0^ at V_g_ ≈ −20 V and the PL spectrum could be described by a superposition of two Lorentzian fittings which center at peak X^0^ and X^−^ respectively as illustrated in Figure 2(b). As the gate voltage goes towards positive values (V_g_ > 0), the free exciton X^0^ gradually diminishes and disappears at Vg > 40. Meanwhile, the red-side X^−^ rises to take over the overwhelming weight of the whole PL until it starts to decrease at V_g_ > 20, probably due to the electrostatic screening effect^28^, and the peak X^−^ is further red-shifted. The electric gating dependence attributes X^−^ to n-type trion (electron-bounded exciton) states. As V_g_ goes to negative bias, the free exciton state X^0^ takes over the weight of the PL and tends to saturate around V_g_ = −70 V. While the trion state X^−^ monotonically diminishes, the redshift also shows a sign of saturation of −34 meV at around V_g_ = −70 V. This confirms the trion (electron-bound exciton) origin of the side band around exciton A in the monolayer transmission spectrum and the trion binding energy of 34 meV in monolayer. If we follow the simplified trion model in conventional quantum wells^17^ and take the effective mass of either *m_e_* = 0.37 and *m_h_* = −0.48^29^ or *m_e_* = 0.27, *m_h_* = −0.32^7^, the binding energy of free exciton is estimated at *E_b_* ≈ 0.34 eV. We will show this estimation is oversimplified in the following part.

Two-photon excitation is a third order optical process involving simultaneous absorption of the two photons, which follows selection rules different from those in one-photon (linear) process. As a photon has an odd intrinsic parity, one- and two-photon transitions are mutually exclusive in systems with inversion symmetry: one-photon transitions are allowed between the states with different parities, while two-photon transitions between the states with the same parity. In systems without inversion symmetry like monolayer TMDCs described by a point group of *D_3h_* symmetry, parity is not a good quantum number and there exist transitions which are both one- and two-photon allowed. Nevertheless, the oscillator strengths of exciton states are generally different between one- and two-photon processes. A simplified exciton model could be described as 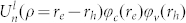, where *φ_c_*(*r_e_*)(*φ_v_*(*r_h_*)) presents the electron (hole) wave function, and 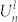 is the function of the relative motion of electron-hole. The optical transition rates for one- and two-photon processes^30^
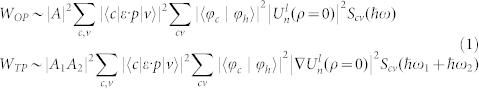
where *A* denotes the vector potential of the excitation, *ε* the light polarization unit vector, 〈*c|ε·p|v*〉 the interband matrix elements, and 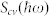 the line-shape function of interband exciton. In a 2D system, 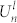 could be described by a solution of 2D Wannier-Mott exciton 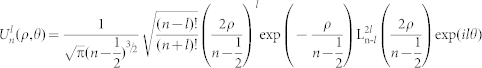 and the exciton binding energy could be described as 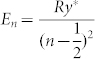, where n = 1,2… is the principle quantum number, *l = 0,1,..(n−1)* is the angular quantum number, and 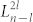 is the associate Laguerre polynomial. As the exciton oscillator strength decays as *n^−3^*, only the ground state (*n = 1*) and the first two excited state (*n = 2*) are considered. In a one-photon process, the ground state *1s* (*n = 1, l = 0*) dominates, whereas in a two-photon process the ground state and *ns* states (*l = 0*) are dramatically subsidized owing to 
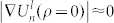
 and the *p* state (*l = 1*) dominates. Hence, the TP-PLE usually reveals the *p*-type excited states of excitons in 2D systems. The one- and two-photon spectroscopy would lead to complementary information on the excitons in monolayer TMDCs.

Figure 3 shows a TP-PLE spectrum of monolayer WS_2 _at ambient condition, where the PL intensity of free band-edge exciton A is recorded as a function of the pulsed excitation energy ranging from 1.18 eV to 1.60 eV (limited by the output of the Ti:sapphire oscillator). With the contrasting optical transition strength, two-photon excitation resonant with the *p*-type excited exciton states dominates while the *s*-type states are nearly invisible. The prominent PL occurs at the excitations around 1.225 eV and around 1.29 eV which are absent in the linear absorption spectrum and considered as p-type states. There is a significant gap state in the range of 1.35–1.365 eV, where the PL intensity drops to be nearly negligible. The negligible but nonzero PL intensity is likely to result from the re-absorption of the second harmonic excitation, since the SHG intensity is higher than that of two-photon luminescence as shown in Figure 3(b). Upon the excitation just above the gap (>1.365 eV) as indicated by the arrow in Figure 3(a), the PL intensity shows a linear increase with the excitation energy (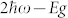) as indicated in the inset. It is the signature of two-photon absorption above the band edge with in-plane polarization in 2D systems^30^,^31^. Besides, the two local minimums at higher excitation energy around 1.44 eV (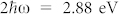) and 1.46 eV (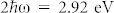) have significant PL intensity; therefore, they are unlikely to be the single-particle band gap state. Thus, the single particle gap or the onset of the interband continuum could be determined at 2.73 eV (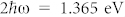). Given the PL peak at 2.02 eV presenting the energy of the ground-state exciton, the exciton binding energy of *E_b_* = 0.71 ± 0.01 eV is extracted from the energy difference between the ground-state exciton and the onset of the inter-band continuum, which is perfectly consistent with Ref. 18.

With the band-edge exciton binding energy of 0.71 eV, we could attribute the peaks around 1.225 eV (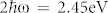) and 1.29 eV (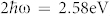) in the TP-PLE spectrum to the excited states of excitons, which are qualitatively consistent with the recent *ab initio* calculation and the experimental findings^18^. As the exciton A and B both originate from the spin-split valence bands at K (K′) valley with the similar effective mass, a similar strength of binding energy is expected. Besides, the PL intensity around the peak A′ and A″ monotonically decreases. Both peaks are likely to be the p-type excited states of the same exciton. Due to the limit of the light source in the experiment, we could not probe the states in the range of 2.0–2.4 eV. If no p-type exciton state appears in the energy of 2.0–2.4 eV, the peak A′ and A″ could be tentatively assigned to the 2p and 3p states of exciton A, respectively. Otherwise, the peak A′ and A″ will be assigned to 3p and 4p states. The exciton binding energy could also be evaluated from the energy difference between exciton *1s* and *np* states. The 2D hydrogen model gives the energy difference between *1s* and *2p* (*3p*)
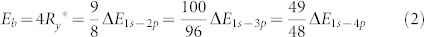
which corresponds to *E_b_* = 0.48 eV and *E_b_* = 0.58 eV, respectively. The alternative assignment, for example, peak A′ and A″ to *3p* and 4*p* states, leads to *E_b_* = 0.45 eV and 0.57 eV (According to Ref. 18, the A′ and A″ are assigned to 3p and 4p excited states of A excitons, respectively).

These are significantly smaller than *E_b_* = 0.71 ± 0.01 eV extracted from the energy difference between the ground state exciton and the onset of the interband continuum The distribution of these excited states also significantly deviates from that of the 2D hydrogen model. The difference may lie in the modification of the 2D hydrogen model by electron-phonon and electron correlation interactions in monolayer TMDCs. The recent first principle simulation shows that q-dependent screening dramatically enhances the binding energy of the excited states of excitons^5^,^18^. Nevertheless, it is safe to extract the exciton binding energy of *E_b_* = 0.71 ± 0.01 eV by the energy difference between *1s* exciton and the onset of the interband continuum, independent of the assignments of the excited states. There exist discrepancies in measurements of exciton binding energy^18^,^19^,^20^,^21^,^22^,^23^,^24^. Besides, controversial results on exciton binding energy of MoSe_2_ monolayers have been reported by scanning tunneling spectroscopy^23^ and angle-resolved photoemission spectroscopy^32^. One possible cause may lie in the difference of samples. A recent theoretical calculation shows that the high doping could significantly modify the exciton binding energy and the optical band gap in monolayer TMDCs^33^.

The two-photon absorption has a quadratic dependence on the excitation intensity in principle. The two-photon induced photoluminescence (TP-PL) intensity from monolayer WS_2_ displays a clear quadratic dependence on the excitation intensity at low excitation intensity as shown in Figure 3(c). As the excitation intensity increases above 0.2 GW/cm^2^, the PL intensity experiences a clear transition from a quadratic dependence to a linear dependence on the excitation intensity. The linear dependence may result from some nonradiative channels arising from the exciton-exciton interactions when the exciton density increases to a certain level. If we follow the simple model
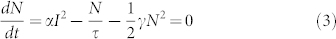
where *N* denotes the exciton density, *I* the excitation intensity, *α* the two-photon absorption cross section, *τ* the exciton lifetime and γ the exciton-exciton annihilation rate, the fitting of the quadratic dependence *I_ph_ = ατI*^2^(*I* → 0) gives two-photon absorption cross section of α ≈ 3.5–5.3 × 10^4^ cm^2^W^−2^S^−1^ at 1.59 eV where the PL quantum yield of 4 × 10^−3^ 9 and the exciton lifetime of 100 ps are assumed^34^. Subsequently, the linear dependence slope at high intensity 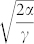 yields the exciton-exciton annihilation rate γ ≈ 0.31–0.47 cm^2^/s, which is qualitatively consistent with that in monolayer MoSe_2_ measured by pump-probe reflection spectroscopy^35^. The linear intensity dependence of TP-PL is the evidence of the strong exciton-exciton interactions in monolayer TMDC.

In summary, the linear absorption spectroscopy cannot resolve the electronic interband transition edge down to 10 K, due to the strong electron-phonon scattering and the overlap of excitons around Γ point and the transfer of the oscillator strength from interband continuum to exciton states. The TP-PLE measurements successfully probe the excited states of the band-edge exciton and the single-particle band gap. The exciton binding energy of 0.71 ± 0.01 eV is extracted by the energy difference between 1s exciton and the single-particle gap in monolayer WS_2_. The distribution of the exciton excited states significantly deviates from the 2D hydrogen model. The giant exciton binding energy manifests the unprecedented strong Coulomb interactions in monolayer TMDCs.

## Author Contributions

X.D.C. conceived the experiments; B.R.Z. and X.C. carried out the experiments and data analysis; All authors discussed and wrote the manuscript.

## Supplementary Material

Supplementary Information Supplementary Information

## Figures and Tables

**Figure 1:**
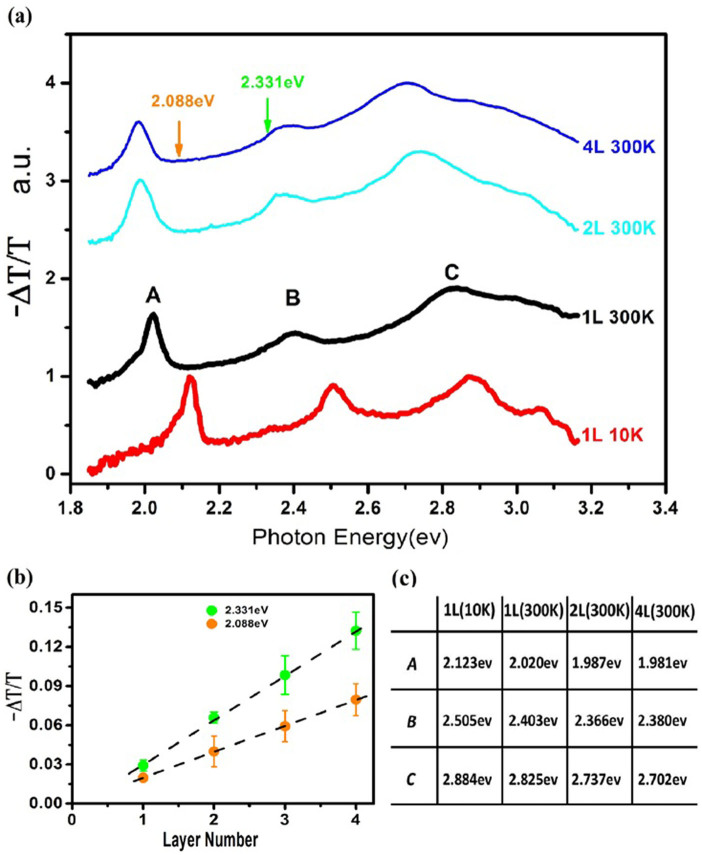
The linear absorption spectra of WS_2_ atomically thin films. (a) Normalized differential transmission spectra of multi- and monolayer WS_2_ at room temperature and 10 K. (b) Absorbance of atomically thin layer at photon energy of 2.088 eV (orange) and 2.331 eV (green) as indicated by arrows in figure 1(a). The absorbance shows a linearly dependence on layer number, each unit layer with constants of 2.0% and 3.4% respectively. (c) Absorption peak energy values of exciton “A”, “B” and “C”.

**Figure 2:**
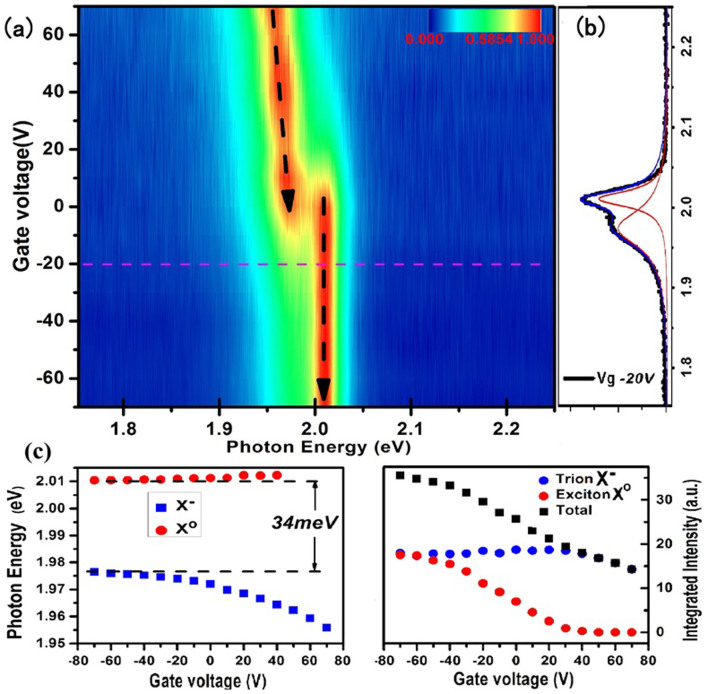
Electric-doping dependent photoluminescence spectra at room temperature in vacuum. (a) Colour contour plot of normalized PL spectra excited by a cw laser (2.331 eV) under various back-gate bias. Dashed black arrows contour PL peaks of free exciton and trion states. Even at zero gate bias the trion *X^−^* exists due to defects or substrate interactions. (b) Illustration of the PL profile at V_g_ = −20 V (dashed line labelled in the top panel) as a superposition of two Lorentzian shape lines in red. (c) Electric gate dependence of excitons′ and trions′ energies (upper panel) and the corresponding integrated intensities (bottom panel).

**Figure 3:**
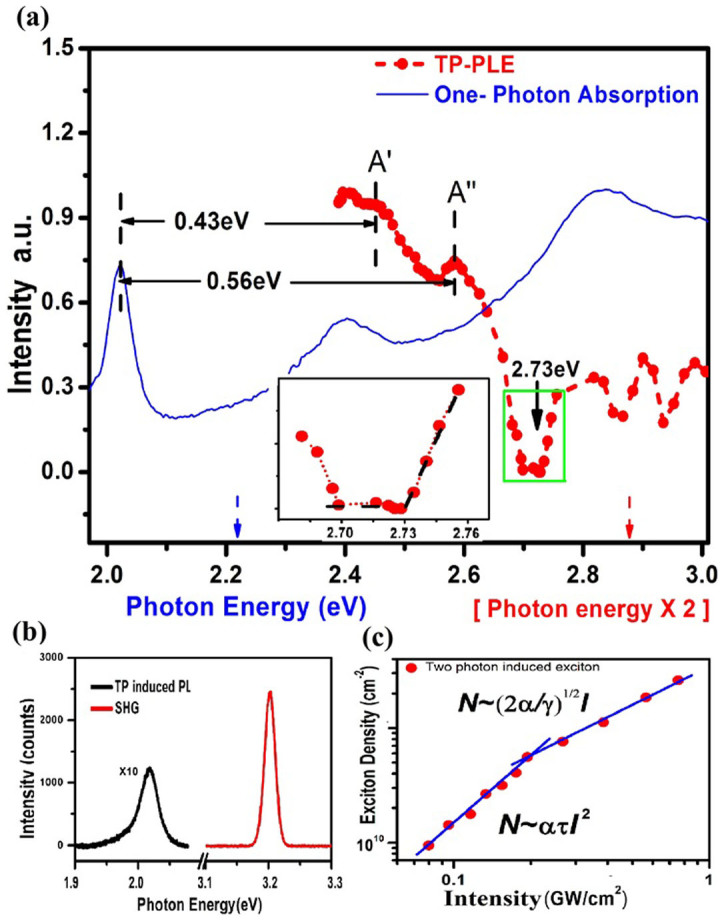
Two-photon photoluminescence excitation spectra at ambient condition. (a) Comparison of one-photon absorption spectrum (blue) in visible range and two-photon PLE spectrum (red) with the excitation in the range of 1.192 ~ 1.5 eV where the x axis presents the exciton energy (in blue) for linear absorption and the double of the excitation energy (in red) for TP-PLE. Both spectra are obtained at ambient conditions. A′ and A″ denote the excited states of exciton A and the zoom-in of the gap state section is shown in inset. The TP-PL intensity linearly increases with the excitation energy just above the threshold of the interband continuum, presenting the signature of two-photon process in 2D systems where the polarization sits in the 2D plane. (b) The spectra of TP-PL and the second harmonic generation (SHG) at the excitation of 1.6 eV. The integrated intensity of the PL is more than one order of magnitude less than that of the SHG. (c) The intensity of ground state exciton vs. the excitation intensity under the excitation of 1.59 eV. The fitting lines demonstrate a quadratic- (under low intensity) and a linear-dependence (under high intensity) respectively, which yield the exciton-exciton annihilation rate γ ≈ 0.31–0.47 cm^2^/s and the two-photon absorption cross section α ≈ 3.5–5.3 × 10^4^ cm^2^W^−2^S^−1^.
