# Supplementary material for: Exciton Binding Energy of Monolayer WS2
Source: Sci Rep. 2015 Mar 18;5:9218. doi: 10.1038/srep09218 (PMC4363839; doi:10.1038/srep09218)
Supplement: Supplementary Information [file srep09218-s1.pdf]

Supplementary Information

Exciton Binding Energy of Monolayer WS_2

Bairen Zhu, Xi Chen, Xiaodong Cui

Physics Department, University of Hong Kong, Hong Kong, China

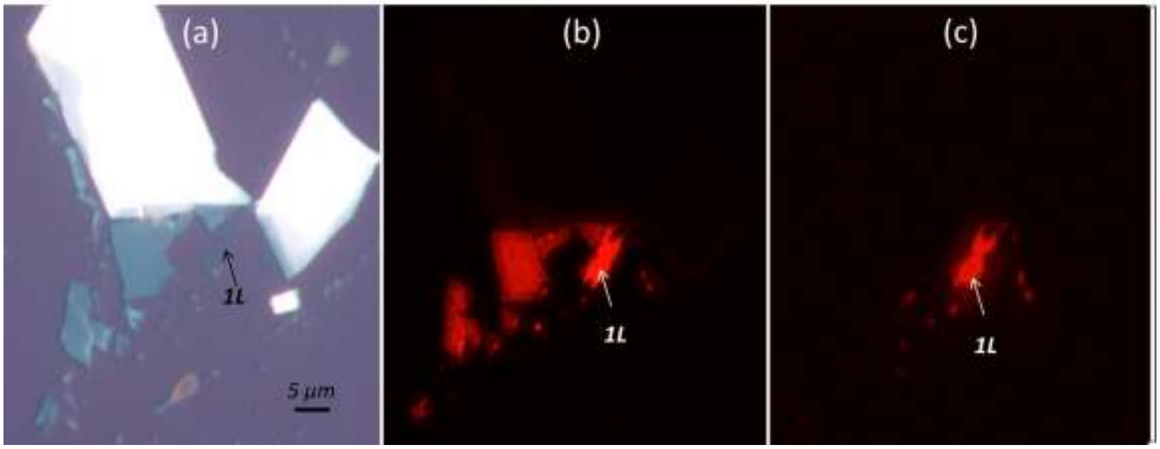

Figure S1. (a) Optical micro-view of WS_2 flakes with a scale bar of 5μm and the monolayer flake marked for clarity. **(b)** Image of second harmonic generation (SHG) shows strongest intensity from the monolayer flake compared to other odd layers, consistent with Ref[1]. **(c)** Mapping of two-photon photoluminescence (TP-PL) indicates only single layer WS_2 has an observable intensity due to its higher quantum efficiency at same condition.

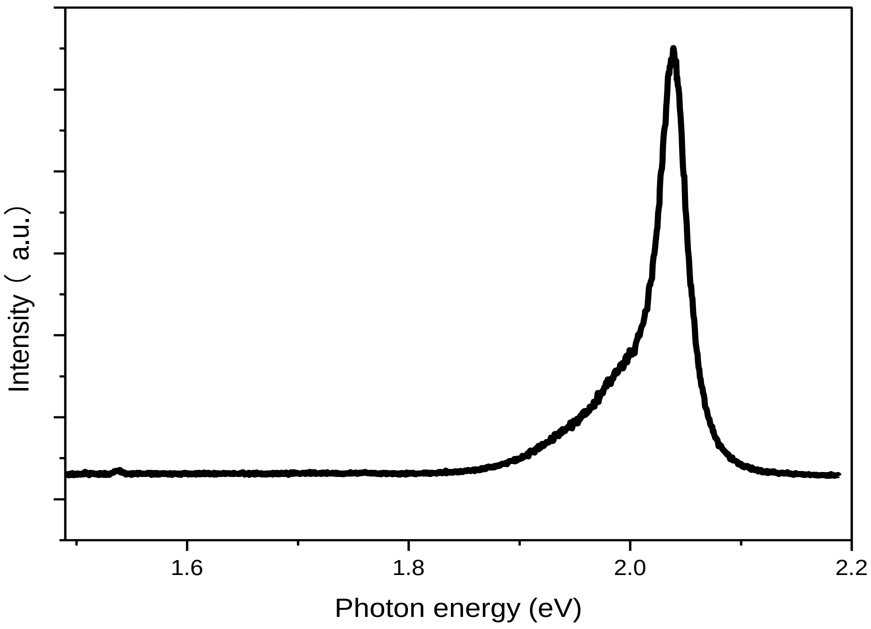

Figure S2. Photoluminescence spectra from monolayer WS_2 excited by 532nm of 1μW and no indirect emission peak found below A exciton, consistent with the nature of direct gap of monolayers. The asymmetric line profile gives a hint of the existence of trion.

Reference

S1. H. Zeng et al., Sci. Rep. 3 (2013)
